# Design, characterization, and evaluation of eco-friendly etofenprox-loaded ethosomes to control *Ceratitis capitata* (Diptera: Tephritidae)

**DOI:** 10.1038/s41598-025-11832-y

**Published:** 2025-07-23

**Authors:** Francesco Corrias, Ines Castangia, Salvatore Marceddu, Roberto Mannu, Arturo Cocco, Maria Letizia Manca, Gabriele Ibba, Maria Manconi, Ignazio Floris, Alberto Angioni

**Affiliations:** 1 Department of Life and Environmental Science, University of Cagliari, University Campus of Monserrato, Cagliari, Italy; 2 Institute of Sciences and Food Production (ISPA), Italian National Research Council (CNR), 07100 Sassari, Italy; 3 Department of Agricultural Sciences, University of Sassari, 07100 Sassari, Italy; 4 Interdepartmental Center IA – Innovative Agriculture, 07041 Alghero, Italy

**Keywords:** Etofenprox, Nanopesticide, Mediterranean fruit fly, Phospholipid-vesicles, Geraniol, Insect pests, Environmental sciences, Nanoscience and technology

## Abstract

*Ceratitis capitata,* is one of the most considerable invasive pests affecting fruit production worldwide. Conventional pesticides are generally formulated using organic solvents and emulsifiers that, in turn, are flammable and toxic. Thanks to their small size and biocompatibility, liposome-like formulations may significantly improve the efficacy and safety of conventional pesticides. This study aims to develop an alternative and innovative etofenprox formulation based on phospholipid vesicles (ethosomes) and evaluate its possible application for agricultural pest control. Ethosomes and geraniol-ethosomes were prepared by the one-step sonication method, achieving vesicles with small sizes (around 267 nm) and low polydispersity index (around 0.04). These vesicles were stable over 90 days of storage at room temperature and could slow the release of etofenprox (57 ± 4% released), in comparison with a commercial formulation (85 ± 5% released) after 24 h. Ethosomes and geraniol-ethosomes showed similar retention properties on lemon leaves (13.3 ± 1.0 to 14.4 ± 1.2 mg/cm^2^) under laboratory condition. After open-field application, geraniol-ethosomes left the highest etofenprox residues on lemon leaves (14.3 ± 1.0 mg/kg), whereas the commercial formulation on the flavedo (4.1 ± 0.5 mg/kg). This result highlighted the possibility of reducing the application dose of etofenprox loaded in geraniol-ethosomes especially during the BBCH stage before fruiting. Both formulations displayed good biocompatibility with no significant cytotoxic effects on human keratinocytes (HaCat cells) across different etofenprox concentrations. Furthermore, laboratory bioassays revealed that geraniol-ethosomes exhibited a prolonged toxicity when sprayed against *Ceratitis capitata* adults, attributed to sustained release kinetics, underscoring their potential in environmentally sustainable agricultural pest management.

## Introduction

The employment of pesticides in agricultural systems is of pivotal importance for the management of plant pests and diseases, with the aim of ensuring optimal production levels. Increased consumer awareness on sustainable agriculture and the adverse effects of insecticides, coupled with the implementation of regulatory policies aimed at reducing the use of broad-spectrum pesticides, have resulted in the withdrawal of several widely used active ingredients in certain countries^1,2^.

*Ceratitis capitata*, also commonly known as the Mediterranean fruit fly, was selected as the target organism for conducting toxicity bioassays. Native to Sub-Saharan Africa, this pest has been accidentally introduced into many fruit-growing areas, including the Mediterranean basin, the Middle East, Western Australia, South and Central America, and Hawaii^3^, being one of the most significant invasive pests affecting fruit production worldwide. *Ceratitis capitata* exhibits a highly polyphagous nature, infesting over 400 wild and cultivated host plant species^4^. The larvae bore into fruit pulp, causing early fruit drop, facilitating the growth of fruit-decaying fungi, and leading to substantial economic losses, which can reach up to 100%. Its ability to infest various hosts and move between them based on fruit ripening periods complicates the pest management, particularly in mixed fruit crop systems, resulting in exponential population growth from early summer to late fall^4^. Conventional methods for controlling *C. capitata* globally rely on synthetic insecticides including etofenprox due to its short pre-harvest interval, which allows its application right before harvest to control vegetable and fruit pests^5^.

Pyrethroids represent a prominent class of pesticides, frequently employed to control pest populations in various environmental settings^6^. These compounds exhibit neurotoxic properties, targeting the voltage-gated sodium channel’s receptor site^7–9^. Despite their high efficiency and low toxicity when compared to other pesticide groups such as organophosphates and carbamates^1^, synthetic pyrethroids are prominent risk contributors in EU streams^10^, especially because they might be very toxic to aquatic organisms and may cause long-term adverse effects in aquatic environments^11^. Due to their lipophilic properties, conventional pyrethroid-based formulations are characterized by large quantities of organic solvents, whose presence led to additional environmental concerns^12^.

Among all pyrethroids, etofenprox is one of the most commercially available, and is used against a wide range of harmful insects. Etofenprox is a non-ester pyrethroid insecticide with a characteristic ether linkage^13^. As with other pyrethroids, its mode of action involves blocking sodium channel ion gates during cell membrane repolarization, thereby interfering with the propagation of nerve impulses along insect nerve axons^14^. Among commercial pyrethroids, etofenprox shows one of the lowest acute toxicities for mammals^15^. Available commercial formulations, such as emulsifiable concentrates, wettable powders, and suspension concentrates, can experience drift and rain fastness after field applications, requiring higher doses and negatively affecting the environment. Moreover, the hydrophobic nature of etofenprox requires the use of formulations with organic solvents and synthetic emulsifiers, which are flammable and potentially toxic for humans as well^16,17^.

An innovative approach to overcome the challenges associated with water-insoluble pesticides involves nanocarriers formulations, ranging from 1 to 500 nm in size, capable of loading and protecting various molecules^18–21^. Nanocarriers offer increased specific surface areas that enhance spreading, adhesion, retention, and accumulation at target sites, although their toxicity, efficacy, and environmental fate remain limited in agriculture. Among these nanocarriers, liposomes are promising due to their simple composition, biodegradability and biocompatibility. These nanometric vesicles consist of one or more concentric bilayers of phospholipids surrounding an aqueous core showing great biocompatibility and biodegradability^22^. The controlled release of etofenprox from conventional liposomes coated with chitosan has already been studied^23^. However, some authors reported that conventional liposomes are not suitable for agricultural applications in complex environments due to their limited stability and tendency toward oxidation and hydrolysis^24^. Therefore, Du et al.^25^ evaluated the performance of a thermo-responsive liposomal dispersion of emamectin benzoate and nitenpyram against the diamondback moth, *Plutella xylostella* (L.) (Lepidoptera: Plutellidae). Chen et al.^26^ prepared and characterized polymeric liposomes for the delivery of emamectin benzoate, whereas Zhang et al.^24^ prepared end evaluated a new cymoxanil non-phospholipid liposomal nanocarrier (i.e., sterosomes). Similarly to liposomes, ethosomes are specific phospholipid vesicles, formulated by adding ethanol (20–45%) in the hydrating medium, and typically used in pharmaceutical formulations for skin applications^27^. In the vesicle structure, ethanol interacts with the polar heads of phospholipids, making the vesicle membrane more flexible than that of liposomes and the carrier more versatile and suitable for different applications^27^.

To the best of our knowledge, no studies have yet explored ethosomes as carriers for the delivery of pesticides in agrochemical applications. Hence, this study aimed to incorporate etofenprox into ethosomes and assess its acute toxicity against the target organisms *Ceratitis capitata* (Wiedemann) (Diptera: Tephritidae) after topical application under laboratory conditions. Geraniol, a monoterpenoid alcohol found in various essential oils, was added to ethosomes to enhance vesicle stability, as it can intercalate into the phospholipid bilayer, improving membrane packing and reducing permeability and fluidity under stress conditions^28^. Liposomes without ethanol and an etofenprox commercial emulsifiable formulation were also tested and used as controls. Phospholipid vesicles were fully characterized in terms of physico-chemical properties (i.e., mean diameter, polydispersity index, zeta potential, stability over time, and morphological structure). The adhesive properties of the formulations correlating the surface tension with data obtained with the leaf retention test were assessed. Additionally, etofenprox residues on lemon flavedo and leaves were quantified after spray application in the open field, along with in vitro cytotoxicity with human keratinocytes (HaCat).

## Materials and methods

### Chemicals and reagents

Granular soybean lecithin and geraniol oil were purchased from Galeno (Carmignano, Italy). Acetonitrile, methanol, and ethanol of analytical grade were purchased from Carlo Erba (Milan, Italy). Etofenprox analytical standard (> 98%) was purchased from Merck (Milan, Italy). Double-deionized water with a conductivity less than 18.2 MΩ was obtained with a Milli-Q apparatus (Millipore, Bedford, MA, USA). QuEChERS reagents Part No: 5982-6650 (4 g magnesium sulfate, 4.1 g sodium chloride, 1 g trisodium citrate dihydrate, 0.5 g disodium hydrogen citrate sesquihydrate) and Part No: 5982-5056 (150 mg primary secondary amine (PSA), 900 mg magnesium sulfate) were from Agilent Technologies (Milan, Italy).

Stock standard solution of etofenprox at 1000 mg/L was prepared in acetonitrile and stored at 4 °C before analysis. Intermediate working standard solutions were prepared daily by dilution with acetonitrile. A commercial etofenprox emulsion concentrate at 280 g/L of active ingredient was purchased in a local market and used as control, whereas etofenprox technical material was kindly provided by the producer. Cell medium, fetal bovine serum, penicillin, streptomycin, fungizone, and all the other reagents for cell studies were purchased from Thermo-Fisher Scientific Inc. (Waltham, MA, US).

### Preparation of etofenprox-loaded phospholipid vesicles

A preliminary formulation study was performed by varying the concentrations of ethanol (0%, 10%, 20%, 40%), geraniol (0%, 4%, 8%, 16%), etofenprox (20, 30, 60 mg/mL), and soy lecithin (30, 60, 90 mg/mL). Each formulation was evaluated in terms of vesicle size, polydispersity index (PDI), zeta potential, and visual appearance, both immediately and after 24 h. The formulation containing 20% ethanol and 8% geraniol was selected as optimal due to its physical stability (no precipitation or phase separation), low PDI, and consistent vesicle size over time.

Ethosomes were then prepared by dispersing etofenprox (30 mg/mL) and soy lecithin (60 mg/mL) in a mixture of double-deionized water (80%) and ethanol (20%) (Table 1). Geraniol-ethosomes were prepared using a hydrating medium composed of double-deionized water (72%), ethanol (20%), and geraniol (8%) (Table 1). Control liposomes were prepared using only double-deionized water as the hydrating medium. All dispersions were then sonicated for twenty cycles (5 s ON, 2 s OFF, 14 µm probe amplitude) using a Soniprep 150 high-performance sonicator (MSE Crowley, London, UK) to obtain small, stable and homogeneous vesicles (Table 1) ^29^. All formulations were prepared in triplicate to ensure reproducibility. Additionally, empty vesicles were prepared as well and used as references.

**Table 1:** Composition of phospholipid vesicles loading etofenprox

Phospholipid vesicles	Soybean lecithin (mg/mL)	Etofenprox(mg/mL)	Ethanol (mL)	Geraniol (mL)	Water (mL)
Liposomes	30	30	–	–	1
Ethosomes	30	30	0.2	–	0.8
Geraniol-ethosomes	30	30	0.2	0.08	0.72

### Physical characterization of phospholipid vesicles

The evaluation of the mean diameter, polydispersity index, and zeta potential of the phospholipid vesicles was performed using a Zetasizer Ultra (Malvern Instruments, Worcestershire, UK). Before analysis, the dispersions were diluted up to the final concentration of etofenprox indicated on the label for open field application (140 mg/L). All samples were analyzed immediately after preparation. Finally, to eliminate the unloaded etofenprox, vesicles (1 mL) were loaded into dialysis tube (Spectra/Por® membranes, 12–14 kDa MW cut-off, 3 nm pore size; Spectrum Laboratories Inc., DG Breda, the Netherlands) and maintained at room temperature (25 ± 1 °C) in 2 L of distilled water for 2 h, refreshing water after 1 h. The concentration of etofenprox was quantified by high-performance liquid chromatography with diode-array detection (HPLC–DAD) after disruption of the vesicles with methanol (1:1000). The incorporation efficiency (%), indicating the percentage of the etofenprox incorporated completely, was calculated as follows^30^:

1 $$Incorporation efficiency = \frac{A \times 100}{B}$$

where A is the concentration of etofenprox before dialysis and B is the initial amount of etofenprox weighed during the preparation step.

### Storage stability of phospholipid vesicles

The shelf-life stability of the prepared phospholipid vesicles was tested by storing them in a closed, dark vial at room temperature for 90 d. Samples were collected at 0, 7, 30, 60, and 90 d, and the mean diameter and polydispersity index were measured. Thermal stability tests were performed according to the Accelerated Storage Procedure (CIPAC MT 46.3) and the Low Temperature Stability of Liquid Formulations (CIPAC MT 39.3). Briefly, the samples were placed in sealed bottles and stored at 54 ºC for 14 d and at 0 ºC for 7 d. At the end of the storage period, the mean diameter, polydispersity index, and external appearance were evaluated.

### Surface tension evaluation

The surface tension of the phospholipid vesicles and the commercial formulation was measured at room temperature using the ring method specified in EEC Method A.5, employing a K20-KRUSS Force tensiometer. Before the measurements, all samples were diluted at 1 g/L of etofenprox, in accordance with SANCO/10,473/2003-rev.5 guidelines (SANCO guidelines). These analyses provide useful information on the ability of a liquid to wet a solid surface such as that of a leaf, fruit, or insect. Low surface tension values correspond to low contact angles and good wetting properties of the liquids.

### Preliminary release study of etofenprox

To simulate and verify the ability of the formulations to release etofenprox , in the first hours after application, a preliminary release study was performed according to Du et al. with minor modification^22^. 1 mL of phospholipid vesicles or the commercial formulation were transferred into polycarbonate dialysis bags (Spectra/Por® membranes: 12–14 kDa MW cut-off, with 3 nm pores; Spectrum Laboratories Inc., USA) and dialyzed against 100 mL of bi-distilled water at room temperature for 24 h. At scheduled time spots (0, 1, 2, 4, 6, and 24 h), 1 mL of the release medium was withdrawn and replaced with an equivalent amount of fresh water. The concentrations of etofenprox in the collected samples were analysed by HPLC–DAD. All analyses were performed in triplicate.

### Leaf retention of formulations

Leaf retention of formulations was assayed on lemon leaves according to Corrias et al.^16^. Briefly, ethosomes, geraniol-ethosomes, liposomes and a commercial formulation (i.e., positive control), were diluted up to 140 mg/L of etofenprox. Water was used as a negative control. Each leaf area (2 cm × 2 cm; n = 5) was weighed using an electronic balance (ABT220-5DM, Kern, Balingen, Germany). Leaves were then completely immersed in the phospholipid vesicle dispersions, commercial formulation or pure water for 10 s, removed and weighed again. Retention (mg/cm^2^) was calculated as:

2 $$Retention = \frac{{W_{1} - W_{0} }}{S}$$

where W_0_ and W_1_ are the leaf weight (mg) before and after the immersion in the samples and S (cm^2^) is the leaf area.

### Open field application

The treatment was carried out on September 1, 2022, in a field located in the municipality of Villacidro (Cagliari, Sardinia, Italy—39.4499589; 8.7640110) on lemon (*Citrus lemon* L.) trees approximately 2 m in height and at the fruiting stage. Tree spacing was 4 m between pairs of rows and 2.5 m between trees in a row. The study included three replicates of the following treatments: (1) water (i.e., untreated control); (2) commercial formulation; (3) ethosomes; (4) geraniol-ethosomes. Samples were sprayed with 2 L of water or formulations containing 140 mg/L of etofenprox using a knapsack sprayer pump (Miura 12—Di Martino SPA, Casoni, Vicenza, Italy). Tree groups were on different rows to avoid drift contaminations. 2 h after application, approximately 4 kg of lemons and 2 kg of leaves were randomly sampled from each treated area, transported to the laboratory, and processed immediately for analysis.

### Sample processing and etofenprox extraction

The lemons were peeled, and the obtained flavedo layers were combined and shredded using a knife, being careful not to break the utricles with the essential oil. A total of 5 g of the homogenized flavedo and 5 g of whole leaves were weighed into a 50 mL test tube, followed by the addition of 20 mL of acetonitrile. The mixture was agitated using a vortex mixer (Reax Top, Heidolph, Germany) for 1 min. Subsequently, 6.5 g of QuEChERS salts (Part No: 5982-6650) were added, and the test tube was agitated for 2 min on the vortex mixer and then for 15 min on a rotary shaker. The sample was centrifuged for 5 min at 4000 rpm and 10 °C (Centrifuge 5810 R, Eppendorf AG, Hamburg, Germany). Then, 5 mL of the supernatant was transferred to a 15 mL test tube containing 1 g of a second QuEChERS salt mixture (Part No: 5982-5056). The tube was agitated for 2 min on the vortex mixer and 15 min on the rotary shaker. The solution was then centrifuged for 5 min at 4000 rpm and 10 °C. Finally, the supernatant was transferred into a 1.8 mL vial for UHPLC analysis. All analyses were performed in triplicate.

#### Determination of etofenprox content

Etofenprox residues after open-field treatment were evaluated using a UHPLC Agilent 1290 Infinity II LC coupled with an Agilent 6470 Triple Quad LC‐MS/MS mass detector with a MassHunter ChemStation. For the other experiments, the quantification was carried out using a HPLC Agilent 1100 series coupled with a photodiode detector (DAD) and a computerized data integration system (ChemStation- Agilent). The instrumental parameters were reported in the supplementary material.

#### Insect culture

Adults belonged from a stock colony maintained at the Department of Agricultural Sciences, University of Sassari (Italy), at 25 ± 2 °C, 60 ± 10% relative humidity (RH), and 14:10 (L:D) photoperiod. Larvae were fed with a diet modified from Ortu et al.^31^ as follows: water (50%), bran (24%), sugar (16.2%), yeast (8.7%), citric acid (0.6%), and benzoic acid (0.5%), while adults were provided with sugar (66.5%), yeast (33.5%), and water ad libitum.

#### Acute toxicity bioassays

Three-day-old female and male Mediterranean fruit fly adults were collected separately in batches of 10 individuals from the colony by an insect aspirator^32^, sedated at − 10 °C for 3 min, and then placed in plastic Petri dishes, 9 cm in diameter, lined with filter paper. The batches of adults were treated with a hand-held sprayer from 20 cm, applying 2.5 mL of each treatment. The tested treatments were: (1) distilled water as a negative control; (2) the commercial formulation as a positive control; (3) empty geraniol-ethosomes; (4) geraniol-ethosomes. The etofenprox commercial formulation and geraniol-ethosomes were applied at the recommended label rate (140 mg/L).

After applications, insects were gently moved to transparent plastic cages (12 × 10 × 3.5 cm) with filter paper on the bottom and a perforated lid. The cage had one hole (0.5 cm in diameter) filled with a cotton plug soaked with 10% sugar solution to feed adults during the experiment. Each treatment was replicated six times (three times each with females and males, respectively). Mortality was checked at 3 and 6 h after application on the first day, and subsequently twice a day until the first treatment reached 100% mortality. The environmental conditions during the experiment were the same as in the *C. capitata* stock colony.

Furthermore, 200 females and males of the same culture were weighted in eight batches of 25 individuals with an electronic balance (Mettler AJ 150, Mettler-Toledo, Columbus, OH, USA), to highlight sex-based weight difference and investigate potential dose-weight responses.

#### Observation of phospholipid vesicles on insects after sprayings

Dispersions of geraniol ethosomes were sprayed onto *C. capitata* adults and the water was allowed to evaporate at room temperature. To analyze the changes that the vesicles underwent during the process, samples were collected, coated with gold/palladium in an Edwards S150A sputter coater unit (Edwards High Vacuum, Crawley, UK), and observed with an environmental scanning electron microscope (Zeiss ESEM EVO LS 10, Jena, Germany) operating at 20 kV in high vacuum mode with a secondary electron detector.

#### In vitro cytotoxicity in keratinocytes

Immortalized human keratinocytes (HaCaT, ATCC collection, Manassas, VA, USA) were grown as monolayers in 150 cm^2^ flasks and incubated with 100% humidity and 5% carbon dioxide at 37 °C. Phenol red-free Dulbecco’s Modified Eagle Medium (DMEM) with high glucose, supplemented with 10% fetal bovine serum, penicillin, streptomycin, and fungizone was used as culture medium. Cells were seeded into 96-well plates (5 × 10^4^ cells/well) and incubated. After 24 h of initial incubation, human keratinocytes (HaCat cells) were exposed for 4 h to etofenprox, either incorporated in ethosomes or dispersed in the commercial formulation, properly diluted with cell culture medium to reach different concentrations of etofenprox (300, 150, 75, 32.5, 16.25, and 8.12 µg/mL). Empty geraniol-ethosomes were used as a control and properly diluted as the other samples. At the end of the experiments, cells were washed with warm phosphate-buffered saline, and their viability was measured using the [3-(4,5-dimethylthiazol-2-yl)-2,5-diphenyltetrazolium bromide] (MTT) colorimetric assay. Briefly, MTT solution dissolved in phosphate-buffered saline (100 µL, 0.5 mg/mL final concentration) was added to each well, and cells were incubated for 3 h. The resulting formazan crystals were dissolved in 100 µL of dimethyl sulfoxide and quantified spectrophotometrically at 570 nm using a microplate reader (Synergy 4, BioTek Instruments, AHSI S.P.A, Bernareggio, Italy). Results are expressed as a percentage of cell viability compared to non-treated cells (100% viability).

In accordance with institutional and national regulations, no ethical approval was required for studies involving commercially available established cell lines.

#### Statistical analysis

Results are expressed as the mean ± standard deviation (SD). Analysis of variance (ANOVA) was used for multiple comparisons of means, and the Tukey’s test and Student’s t-test were performed to test for differences between groups using GraphPad Prism 9. The differences were considered statistically significant for *p* < 0.05.

Regarding the acute toxicity bioassay, statistical analysis was performed using R software, version 4.3.2^33^. The efficacy of tested treatments was corrected over control mortality by the Schneider Orelli’s formula^34^:

3 $$M_{xt} = \frac{{{\Delta }M_{xt} - \overline{M}_{Ct} }}{{100 - \overline{M}_{Ct} }} \times 100$$

where $\Delta {M}_{xt}$ is the adult mortality in treatment *x* after *t* hours, and ${\overline{M} }_{Ct}$ is the average adult mortality *t* hours after the applications (HAA) in untreated control. Percent mortality data were arcsine square root transformed before the analysis to meet the assumptions of normality. Differences in mortality among treatments were evaluated separately within sampling times (i.e., 24, 48, 72, 96, 120 h after application) using a two-way Analysis of Variance (ANOVA) with treatment, sex, and their interaction as fixed factors. Fixed factors and their interaction were compared by Tukey’s test (*p* < 0.05). The weight of *C. capitata* adults was also compared between sexes using ANOVA.

Survival of treated Mediterranean fruit fly was analysed by a mixed effects Cox proportional hazard model using “survival”, and “coxme” packages^35^ in R software. Survival model was fitted considering treatments and sex as fixed factors and cage (i.e., replicate) as a random effect factor. The statistical significance of differences in survival was evaluated using post hoc analysis (*p* < 0.05) with a Bonferroni correction for multiple testing using the “multcomp” package in R software^36^.

## Results and discussion

### Preparation and physical characterization of etofenprox-loaded phospholipid vesicles

In the present study, phospholipid vesicles were prepared using the one-step sonication method. Different ratios of lecithin, ethanol, geraniol oil, and etofenprox were tested to select the most stable vesicles with a small size and low polydispersity index. The sonication energy was modulated by varying the number of cycles (between 5 and 40) and the amplitude (12 to 14 μm) to find out standardized and reproducible conditions capable of producing monodisperse and homogeneous samples upon visual inspection. Basic/conventional liposomes were highly unstable, the dispersion broke down to form a two-phase system, and etofenprox precipitated within 24 h, so these samples were not further characterized and considered unsuitable for the study. The addition of ethanol (20%) enabled the formation of more homogeneous and stable vesicular dispersions. At this concentration, ethanol also contributed to the eco-friendly profile of the formulation, being a volatile, biodegradable solvent with low toxicity, commonly used in pharmaceutical and agricultural applications^37^. It represents a safer alternative to synthetic organic solvents typically found in conventional pesticide formulations. However, despite its stabilizing effect, the system’s capacity to incorporate high amounts of insecticide remained limited, resulting in partial precipitation of etofenprox. Given that etofenprox is a highly lipophilic compound, with an octanol–water partition coefficient (log P) of 6.9 at 20.1 °C, geraniol was added to the ethosomal system due to its lipid-solubilizing properties. The inclusion of geraniol significantly improved the physicochemical characteristics of the formulation, likely promoting better incorporation and stabilization of the insecticide within the lipophilic bilayer of the vesicles. Ethanol and geraniol contributed synergistically to vesicle stability through complementary effects. Ethanol, a key component of ethosomal systems, interacts with the polar headgroups of phospholipids, increasing membrane fluidity and elasticity^37^. This effect reduces vesicle aggregation and enhances drug entrapment efficiency. Geraniol, a lipophilic monoterpenoid, integrates into the phospholipid bilayer, promoting tighter lipid chain packing and reducing membrane permeability. These combined effects enhance the structural integrity of the vesicles and prevent the precipitation of lipophilic etofenprox during storage^38^. Notably, no precipitation of etofenprox was observed throughout the storage period. All vesicular formulations were prepared without insecticide as well, to evaluate its effect on vesicle structure and assembling. The mean diameter of empty control liposomes was 53 ± 1 nm, the polydispersity index was 0.11 ± 0.01, and the zeta potential was − 44 ± 2 mV. The addition of ethanol in empty ethosomes led to a slight increase in size, up to 61 ± 2 nm, but still within a small size range. The addition of geraniol in empty geraniol-ethosomes caused a higher increase on mean diameter (119 ± 7 nm), polydispersity index (0.26 ± 0.02), and zeta potential (− 53 ± 1 mV), confirming its impact on vesicle assembly (Table 2). The loading of etofenprox significantly increased the mean size of vesicles in a way opposite to that of empty vesicles, as in this case liposomes were the larger (300 ± 9 nm, *p* < 0.05 versus other values), followed by ethosomes (279 ± 5 nm, *p* > 0.05 versus that of geraniol-ethosomes) and by geraniol-ethosomes (267 ± 6 nm, *p* > 0.05 versus that of ethosomes), which were the smallest (Table 2). The polydispersity index of etofenprox loaded-geraniol-ethosomes was the lowest (0.04 ± 0.002, *p* > 0.05 versus ethosomes values), confirming a high uniformity of these dispersions irrespective of their large size (Table 2), whereas control liposomes and ethosomes were slightly polydisperse with 0.30 and 0.13 polydispersity index, respectively. All vesicles, irrespective of their composition, had highly negative charges, which may enhance dispersion stability due to the high electrostatic repulsion between vesicles. Specifically, empty geraniol-ethosomes had the highest zeta potential (− 53 ± 1 mV), which decreased to − 42 ± 1 mV after the addition of the insecticide (Table 2). This decrease in zeta potential could likely be due to the incorporation of the lipophilic etofenprox within the phospholipid bilayer, which may partially mask the negatively charged phosphate groups at the vesicle surface, reducing the surface potential^39^. Additionally, except for control liposomes that did not load enough amount of etofenprox, tested vesicles loaded high amount of insecticide: ethosomes 85 ± 3% and geraniol-ethosomes 97 ± 6% (*p* > 0.05 between the two values), highlighting the critical role of the oil in retaining etofenprox within the vesicles (Table 2). Overall, the data suggest that geraniol-ethosomes have suitable characteristics, such as stability, size uniformity, and insecticide incorporation, that make them a promising formulation for delivering etofenprox. The commercial formulation, after dilution following the label recommendation, formed oil droplets in the nanometric range (228 ± 12 nm, p < 0.05 versus other values), which were however highly polydisperse (PI 0.35 ± 0.08, *p* > 0.05 versus empty liposomes, etofenprox-loaded ethosomes, and geraniol-ethosomes) (Table 2). In this study, for the first time, ethosomes loaded with pesticide were prepared. Du et al.^22^, prepared emamectin benzoate-loaded liposome nano-vesicles using lecithin and cholesterol and obtaining vesicles smaller than our geraniol ethosomes (110 nm vs 267 nm) but less homogeneously dispersed (0.272 vs 0.04). Also, Chen et al.^26^ prepared emamectin nanoparticles, but composed of DSPE-PEG2000-NH_2_. The hydrodynamic diameter of their particles accounted for 118 nm. Finally, Hwang et al.^23^ prepared nano–sized chitosan carrier loaded with etofenprox showing an average size of about 800 nm after SEM analysis.

**Table 2:** Mean diameter (nm), polydispersity index (PI), zeta potential (ZP, mV), and incorporation efficiency (E) of empty and etofenprox-loaded phospholipid vesicles.

Phospholid vesicle	Mean diameter (nm)	PI	Zeta potential (mv)	E (%)
Empty liposomes	53 ± 1^a^	0.11 ± 0.01^a^	− 44 ± 2^a^	–
Empty ethosomes	61 ± 2^b^	0.27 ± 0.02^b^	− 40 ± 1^a^	–
Empty geraniol-ethosomes	119 ± 7^c^	0.26 ± 0.02^b^	− 53 ± 1^c^	–
Liposomes	300 ± 9^f^	0.30 ± 0.07^b^	− 41 ± 6^ab^	–
Ethosomes	279 ± 5^e^	0.13 ± 0.08^a^	− 47 ± 1^b^	85 ± 3^a^
Geraniol-ethosomes	267 ± 6^e^	0.04 ± 0.01^a^	− 42 ± 1^a^	97 ± 4^b^
Commercial formulation	228 ± 12^d^	0.35 ± 0.08^b^	–	–

Mean values ± standard deviations are reported (n = 6). For each variable, different letters indicate statistically different values (*p* < 0.05).

### Storage stability

**Fig. 1:**
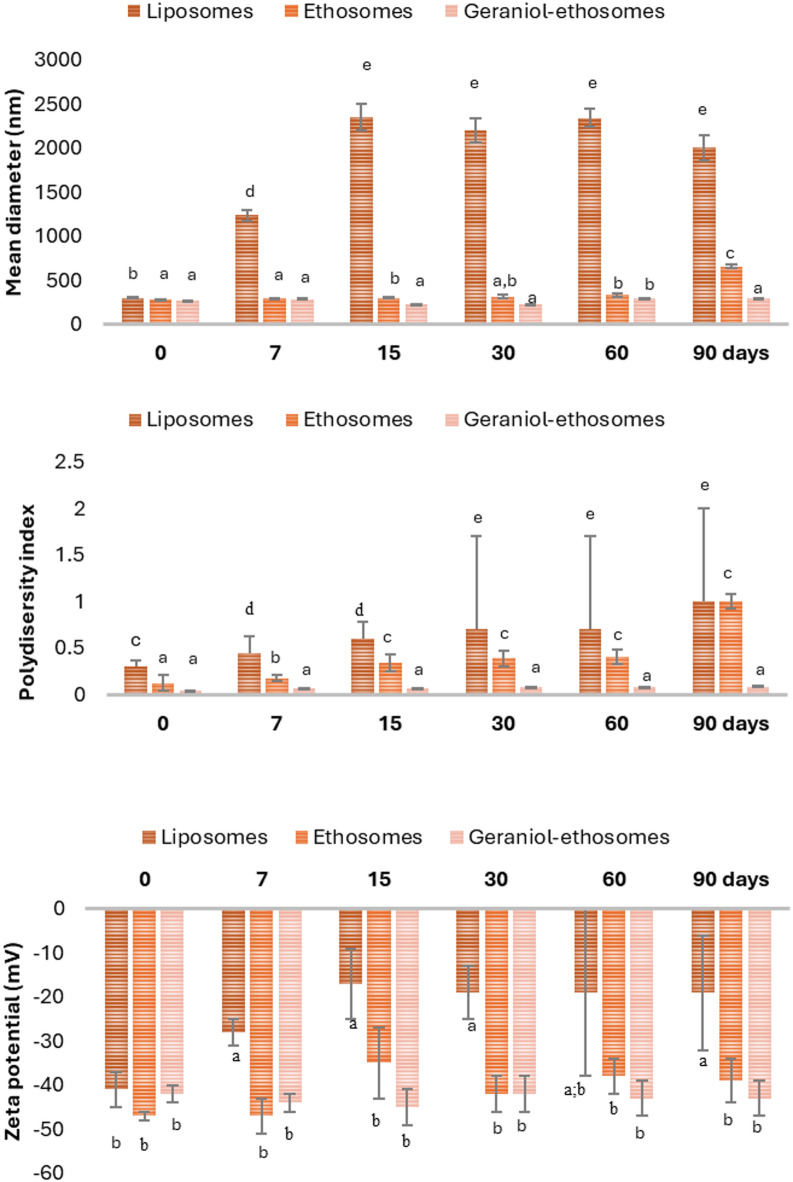
Assessment of storage stability of liposomes, ethosomes and geraniol-ethosomes stored for 90 days at room temperature: (**A**) Mean diameter; (**B**) Polydispersity index; (**C**) Zeta potential. Mean values ± standard deviations are reported (n = 3). For each group (mean diameter, polydispersity index, zeta potential), the same letter indicates values that are not statistically different from each other (*p* > 0.05).

The stability of the phospholipid vesicles over time was evaluated storing the dispersions at room temperature and measuring their size, polydispersity index, and surface charge during the storage at scheduled intervals up to 90 d (Fig. 1). Increased values of the mean diameter and the polydispersity index of control liposomes were observed at 7 d, confirming the system breakdown (Fig. 1). Although ethosomes maintained a consistent mean diameter at 15 d, the polydispersity index exceeded 0.3, indicating a possible reorganization of the vesicles in a non-homogeneous dimensional population. Dispersion of geraniol-ethosomes were the most stable as the tested parameters remained unchanged during all storage period (Fig. 1). Considering the better behavior of geraniol-ethosomes, the dispersion was stored at 54 °C for 14 d and their physico-chemical characteristics were measured after this thermal stress. At this temperature, the mean diameter and the polydispersity index of geraniol-ethosomes increased up to 386 ± 6 nm and 0.34 ± 6, respectively (Table 3), probably because the elevated temperature increased the kinetic energy of the vesicles, promoting their collisions, breaking, and partial reaggregation into larger and less uniform structure.

**Table 3:** Mean diameter, polydispersity index, zeta potential of geraniol-ethosomes after accelerated test.

Test type	Mean diameter (nm)	Polydispersity index	Zeta potential (mV)
After 14 days at 54 °C	386 ± 6^a^	0.34 ± 0.02^a^	− 42 ± 1^a^
After 7 days at 0 °C	278 ± 7^b^	0.08 ± 0.01^b^	− 44 ± 1^a^

Mean values ± standard deviations are reported (n = 3). For each variable, the same letter indicates values that are not statistically different from each other (*p* > 0.05).

Our findings contrast with what reported by Du et al., after 14 days at 54 °C, they observed a decrease in the mean size of their formulations of about 11%^22^. This behaviour was already observed by Hu et al.^40^ after storing zein-alginate core/shell nanoparticles at 90 °C; the mean particle diameter of the nanoparticles was insensitive to thermal processing.

The ability of geraniol-ethosomes to reassemble after thermal shock was evaluated as well. This treatment did not induce statistically significant changes of their size and polydispersity index, that were comparable to those measured before the treatment, confirming a viable shelf-life for agricultural use (Table 3).

### Surface tension evaluation

Considering the better stability of ethosomes and geraniol-ethosomes, these phospholipid vesicles were extensively analysed. The measured surface tensions were lower than that of the water (70.00 ± 0.05 mN/m), which was used as a negative control (Table 4). The commercial formulation had the lowest surface tension (38.30 ± 0.09 mN/m), followed by geraniol-ethosomes (41.17 ± 0.10 mN/m) and ethosomes (48.35 ± 0.03 mN/m) (Table 4).

**Table 4:** Surface tension (γ) and retection of ethosomes, geraniol-ethosomes, commercial formulation and water.

Target	γ (mN/m)	Retention (mg/cm^2^)
Ethosomes	48.35 ± 0.03	14.4 ± 1.0
Geraniol-ethosomes	41.17 ± 0.10	13.3 ± 1.2
Commercial formulation	38.30 ± 0.09	13.8 ± 1.2
Water	70.00 ± 0.05	7.1 ± 0.23

Mean values ± standard deviations are reported (n = 10).

The low surface tension of the commercial formulation was due to the high content of emulsifiers and surfactants, that allow the stabilization of the emulsion after dilution with water before the use. Ethanol, which has a surface tension of approximately 22.0 mN/m at 20 °C, significantly influenced the properties of ethosomes and geraniol-ethosomes. According to SANCO/10,473/2003-rev.5 guidelines, formulations with a surface tension below 60 mN/m are classified as surface-active products (SANCO guidelines).

### Preliminary release study of etofenprox

**Fig. 2:**
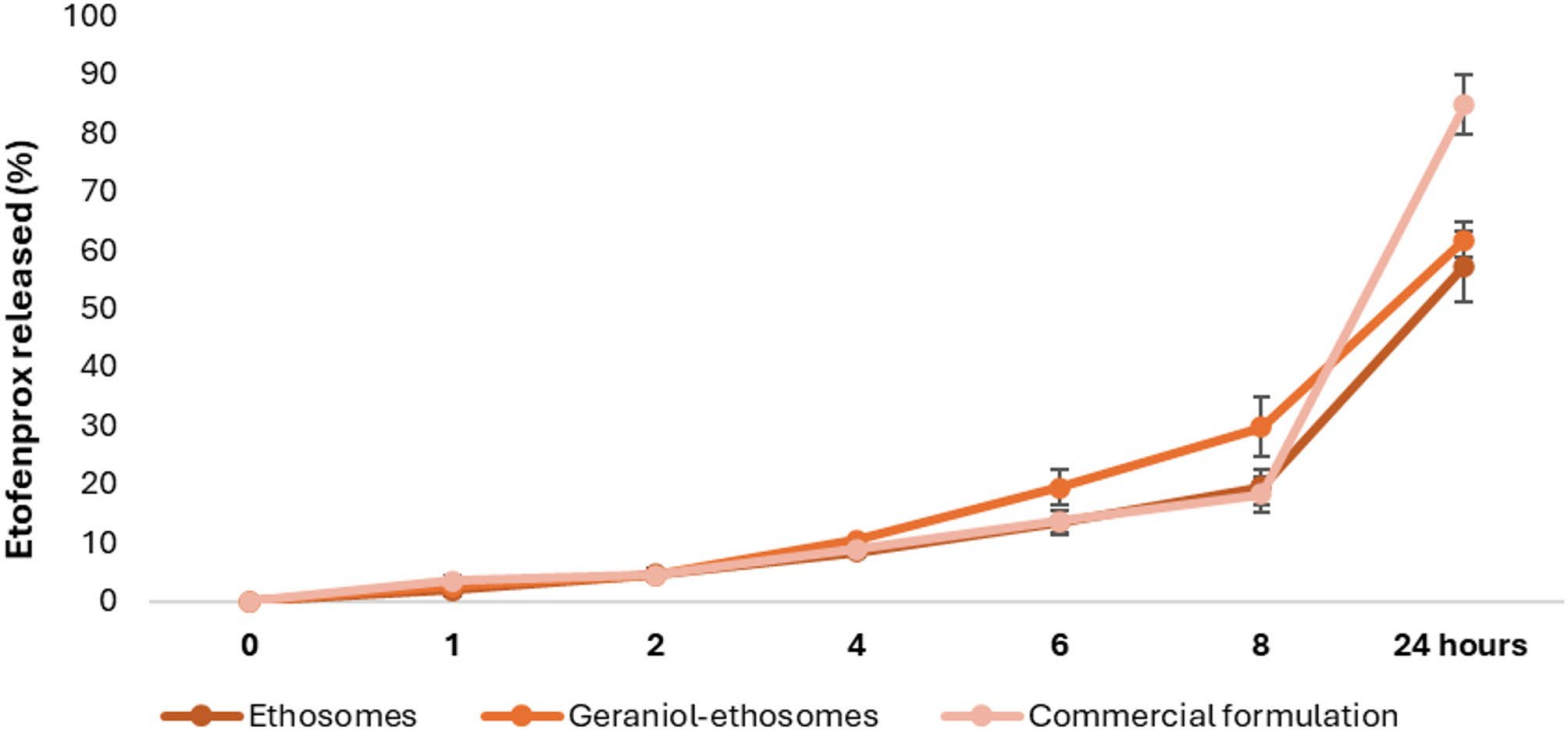
In vitro release of etofenprox (%) from ethosomes, geraniol-ethosomes and commercial formulations over 24 h at room temperature. Mean values ± standard deviations are reported (n = 6).

A preliminary in vitro release study was performed to evaluate the ability of the ethosomes and geraniol-ethosomes to control the release of etofenprox in the first 24 h after application. The liquid commercial emulsifiable formulation was used as control. At 1, 2, and 4 h, the amount of etofenprox released either from ethosomes, geraniol ethosomes, or commercial formulation was low and similar (< 10%). At 6 and 8 h, the amount of payload released from ethosomes and commercial emulsifiable formulation was still equal and low (≤ 10%), whereas that from geraniol-ethosomes was slightly higher (≤ 20%) (Fig. 2). At 24 h, the amount of etofenprox released from the commercial formulation was the highest (85 ± 5%), whereas that released from ethosomes was only 62 ± 6% and that from geraniol-ethosomes was 57 ± 4%, confirming the higher ability of vesicles, especially geraniol-ethosomes, to control and slow down the release of the loaded pesticide. The use of these formulations in agriculture is expected to extend the permanence and the release of pesticide on the target surface by reducing the frequency of its application. Hwang et al.^23^ evaluated the release of etofenprox from chitosan-coated liposomes obtaining a slower release rate in comparison with that provided by ethosomes and geraniol-ethosomes, probably due to the presence of chitosan surrounding the bilayer of the vesicles. However, further release studies will have to be performed considering a longer time.

### Leaf retention of formulations

The deposition rate and adhesive strength of formulations on the leaves play a pivotal role in decreasing pesticide loss due to rolling down and run-off^16^. Therefore, the retention of ethosomes, geraniol-ethosomes and commercial formulation was measured (Table 4). After their application on lemon leaves, the commercial formulation together with phospholipid vesicle dispersions had similar retention values, ranging from 13.3 ± 1.1 mg/cm^2^ (geraniol-ethosomes and commercial formulation) to 14.4 ± 1.0 mg/cm^2^ (ethosomes), which was always higher than that of water (7.1 ± 0.2 mg/cm^2^), used as a negative control.

### Open field insecticide application

**Fig. 3:**
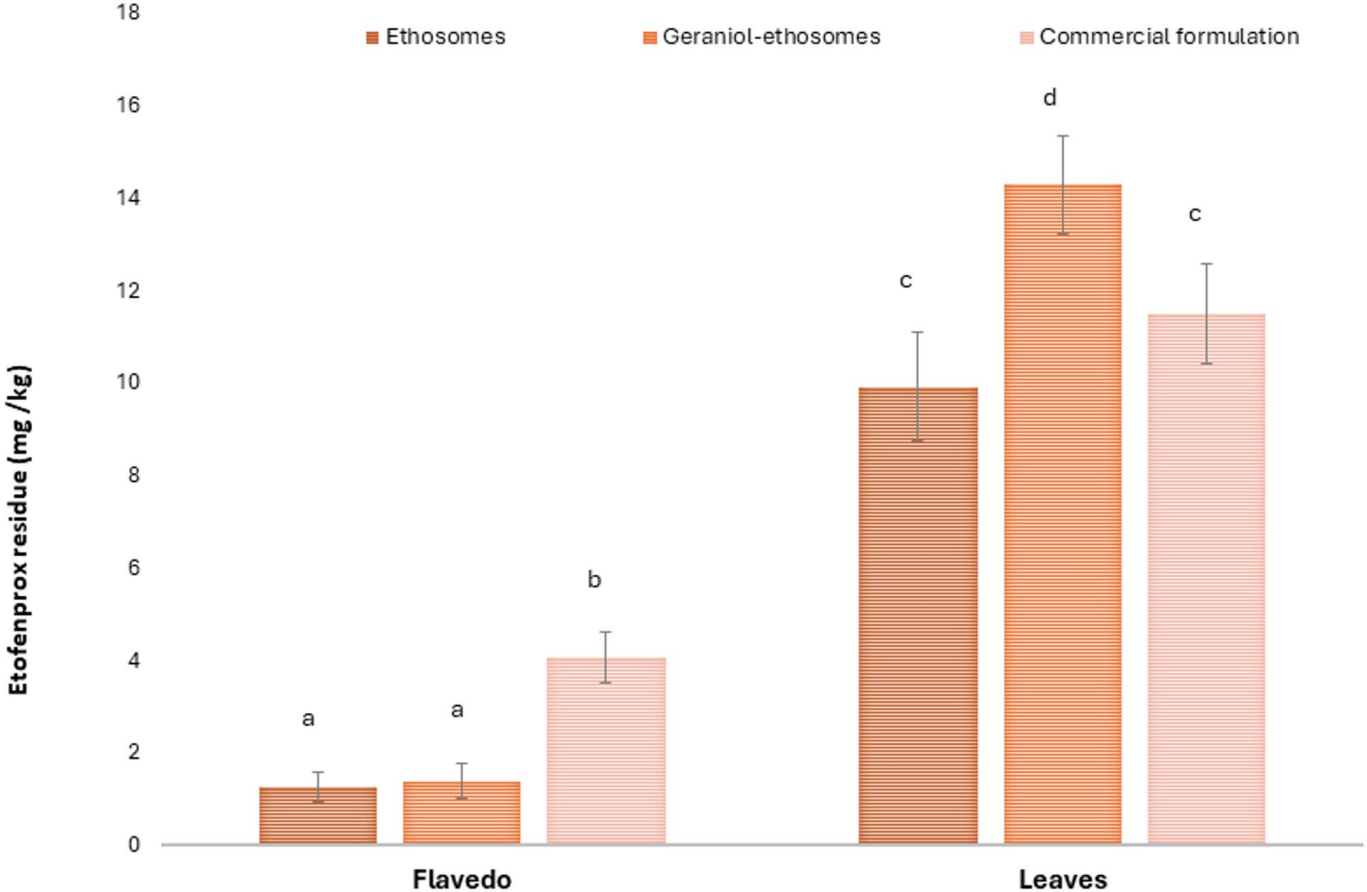
Etofenprox residues (mg/kg) on leaves and flavedo after open field application of the ethosomes, geraniol-ethosomes and commercial formulation. Mean values ± standard deviations are reported (n = 3). For each variable, the same letter indicates values that are not statistically different from each other (*p* > 0.05).

Lemon trees were sprayed with etofenprox formulations and the residual concentrations on both leaves and flavedo were quantified (Fig. 3). After open-field treatment with ethosomes, the residual concentration of etofenprox was 9.9 ± 1.2 mg/kg on leaves and 1.2 ± 0.3 mg/kg on flavedo, whereas it was 14.3 ± 1.0 mg/kg on leaves and 1.4 ± 0.4 mg/kg on flavedo after exposure with geraniol-ethosomes, and 11.5 ± 1.1 mg/kg on leaves and 4.1 ± 0.5 mg/kg on flavedo using the commercial formulation. No traces of etofenprox were found in the negative control treated exclusively with water. According to the literature, residues on leaves were always higher than those on the fruits, probably because of the larger surface area of leaves in comparison with fruits even considering the same weight^16,41^. Moreover, during field application, the fruits are usually protected from the spray by the foliage^42^.

Geraniol-ethosomes left higher etofenprox residues on leaves compared to ethosomes and the commercial formulation (*p* < 0.05 versus other values), whereas the higher etofenprox concentration on flavedo was found after the application of the commercial formulation (*p* < 0.05 versus other values) (Fig. 5). Several factors can influence the distribution of pesticides on the plant, including the physical properties of the used formulation and the distinctive characteristics of leaf and fruit surfaces, such as macro- and micro-roughness and the different epicuticular waxes^43,44^. Considering the same dimensional range (Table 2) and a similar retention ability (Table 4) of the tested formulations, data obtained in the field treatment were influenced by the nature of the plant protection product. Ethosomes and geraniol-ethosomes are solid dispersions of phospholipid vesicles in water, whereas the commercial formulation, after dilution with water, consists of an oil-in-water nano-emulsion. Upon impacting the leaf surface, spray droplets tend to spread or shatter, whereas solid particles typically rebound and are subsequently either retained or rejected^43^. Thanks to their increased specific surface area, solid nano particles were able to cover uniformly the leaves and fill the natural depressions present in the leaves of plants leading to an increase in pesticide residue^16^. Regarding fruits, after recoiling, the spherical shape of the fruit probably promoted the fall off of the larger vesicles from the flavedo. On the contrary, the better wetting capacity of the commercial formulation (Table 4), coupled with the presence of surfactants and adhesive co-formulants, led to a higher residue of etofenprox on flavedo.

However, most of the studies concerning nano-pesticide application are focused on technological or microbiological aspects. The articles in the literature that explore the influence of nanotechnologies on pesticide residues on plants in real field conditions are very limited. More in-depth studies will need to be carried out to better understand the interaction of nanotechnologies on leaves and flavedo after application in open fields.

### Mediterranean fruit fly acute toxicity bioassay

**Fig. 4:**
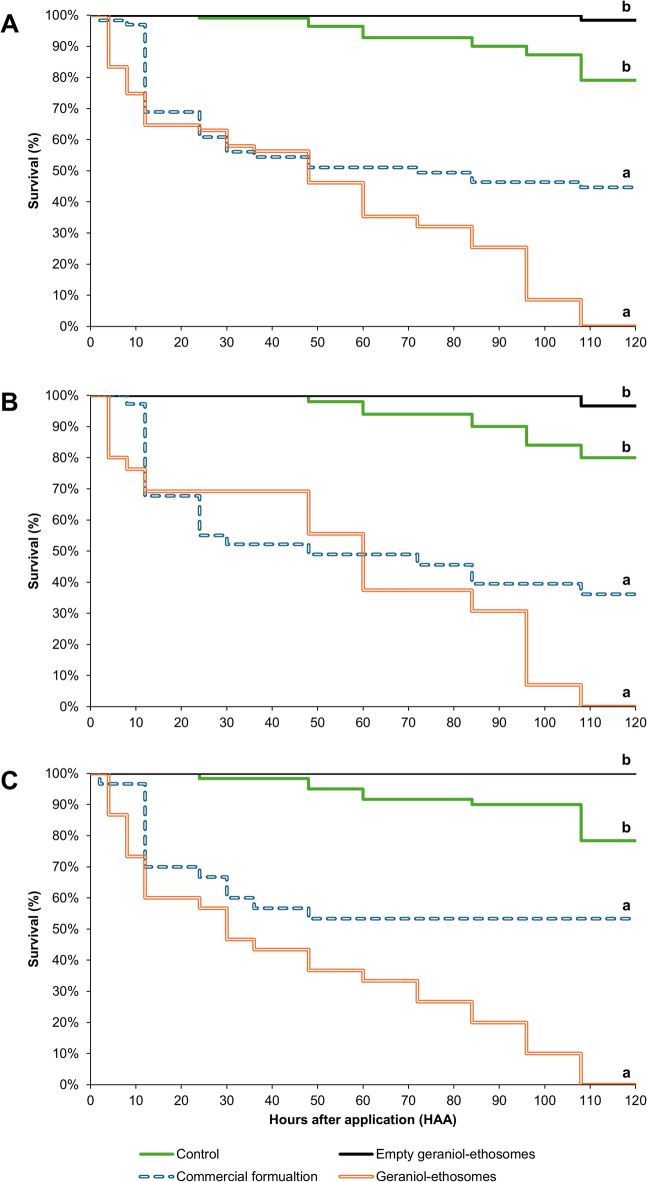
Survival pattern of *Ceratitis capitata* adults by direct application of different etofenprox formulations. (**A**) Males and females. (**B**) Males. (**C**) females. Letters on the right of survival curves indicate a significant difference by Cox proportional hazard model (*p* < 0.05).

The survivorship of *C. capitata* adults was found to be significantly influenced by treatments (Cox model: χ^2^ = 60.051; *p* < 0.001) (Figs. 4A, B, and C). The effect of the treatments was significant during the entire experiment since 12 HAA (Table 5). The values of survivorship after the application of the commercial formulation and the geraniol-ethosomes were not statistically different. The patterns were initially similar, as survival decreased steeply at 12 HAA and slightly at 48 HAA (51% and 46%, respectively). Afterwards, survival of adults treated with the commercial formulation was almost constant (45% at 120 HAA), whereas adult survivorship treated with the geraniol-ethosomes decreased to 0% at 120 HAA. The survival patterns following the application of distilled water and the empty geraniol-ethosomes were not significantly different and the cumulative survivorship at the end of the bioassay (at 120 HAA) was 79% and 98%, respectively (Fig. 4A). These data fit with the result obtained in the release study. Indeed, after 24 h, the commercial formulation released all the etofenprox (about 80%), supplying an unnecessary dosage of pesticide to Mediterranean fruit flies in the first day and limited toxic effect in the following days (Fig. 4A). On the contrary, the sustained release of etofenprox ensured by the geraniol-ethosomes (50% of etofenprox content after 24 h) allowed a prolonged supply of the pesticide to *C. capitata* adults throughout the test thereby leading to increasing mortality rate up to 100% after 5 d. Overall, the fixed factor “sex” did not significantly affect the adult survival (Cox model: χ^2^ = 0.110; *p* = 0.740), although the weight was significantly higher in females (mean ± SE = 9.659 ± 0.057 mg) than in males (mean ± SE = 7.933 ± 0.075 mg) (*F*_1,14_ = 297.40; *p* < 0.001). Accordingly, sex and the interaction treatment*sex were not significant during the experiment (Table 5). The survivorship of males and females exposed to the experimental treatments displayed patterns like the combined pattern, with survivorship due to the commercial formulation and the geraniol-ethosomes being significantly lower than those of males and females sprayed with empty geraniol-ethosomes and distilled water (Fig. 4B and 4C). The incorporation of active ingredients in liposome-like nanocarriers has been proven to increase both the insecticidal effects and duration of residual activity of the formulation^45^. Concurrently, such nanocarriers facilitated controlled-release and co-delivery pesticide systems, as evidenced by *P. xylostella* larvae fed with fresh cabbage leaves treated with emamectin benzoate-loaded liposome nano-vesicles^25^. The temporal pattern of Mediterranean fruit fly survival after application of geraniol-ethosomes under laboratory conditions was found to be comparable to that observed for females of the two-spotted spider mite, *Tetranichus urticae* (Koch) (Acari: Tetranychidae), exposed to abamectin-loaded liposomes with LC_30_ concentrations of the designed formulations, which showed a regular increase of pest mortality over time^46^.

**Table 5:** Effect of experimental factors (i.e., sex and treatment) and their interaction on mortality of *Ceratitis capitata* adults at 24, 48, 72, 96, and 120 h after application (HAA).

Factor or interaction	Statistics (*F*, d.f., p)^a^					
	12 HAA	24 HAA	48 HAA	72 HAA	96 HAA	120 HAA
Treatment	***F***_**3,21**_** = 21.07**	***F***_**3,21**_** = 14.96**	***F***_**3,21**_** = 19.94**	***F***_**3,21**_** = 17.11**	***F***_**3,21**_** = 21.24**	***F***_**3,21**_** = 27.26**
	***P* = < 0.001**	***P* = < 0.001**	***P* < 0.001**	***P* < 0.001**	***P* < 0.001**	***P* < 0.001**
Sex	*F*_1,21_ = 0.01	*F*_1,21_ = 0.01	*F*_1,21_ = 0.38	*F*_1,21_ = 0.01	*F*_1,21_ = 0.16	*F*_1,21_ = 0.69
	*P* = 0.935	*P* = 0.944	*P* = 0.544	*P* = 0.962	*P* = 0.697	*P* = 0.417
Treatment*sex	*F*_3,16_ = 0.01	*F*_3,16_ = 0.19	*F*_3,21_ = 0.76	*F*_3,21_ = 0.15	*F*_3,21_ = 0.46	*F*_3,16_ = 0.25
	*P* = 0.993	*P* = 0.904	*P* = 0.530	*P* = 0.928	*P* = 0.710	*P* = 0.862

^a^Linear model, significant effect at *p* < 0.05.

The use of empty geraniol-ethosomes did not result in any toxic effects in adult Mediterranean fruit flies (Fig. 4). Our findings are in contrast with previous studies in which nanocarriers with geraniol as the active ingredient tested against several horticultural and stored-product pests displayed high incorporation efficiency, long-lasting stability, and high pest toxicity^47^. However, despite the documented toxicity of geraniol to insects, most research has been focused on the assessment of its larvicidal properties, particularly for mosquitoes. With regard to Diptera, geraniol was found to explicit a repellent effect on *Drosophila suzukii* adults^48^, whereas no data concerning its toxicity towards *C. capitata* adults has been reported in the literature. Specifically, geraniol was found to exert a toxicity effect to other insects such as *Cimex lectularius*, as well as to *Bemisia tabaci* when applied as a fumigant^49,50^. Further studies are needed to evaluate the ability of the new formulations to control fruit flies in open field conditions.

### Ability of phospholipid vesicles to deposit on flies

**Fig. 5:**
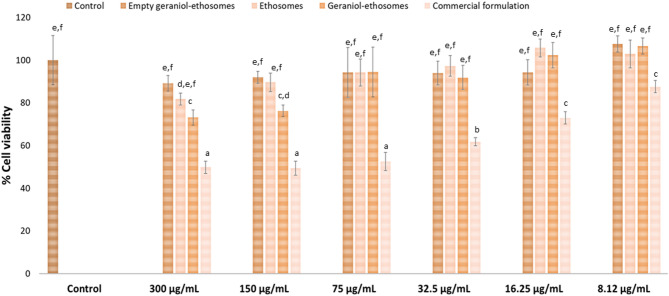
Viability of keratinocytes (HaCat) incubated for 4 h with ethosomes, geraniol-ethosomes, and the commercial formulation diluted in the cell medium to reach 300, 150, 75, 32.5, 16.25, and 8.12 μg/mL of etofenprox. Mean values ± standard deviations are reported (n = 8). Different letter indicates values that are statistically different (*p* < 0.05).

Geraniol-ethosomes were sprayed on flies, coated and observed using an environmental scanning electron microscope (Fig. 5). The coated vesicles, visible on the support surface, had a spherical shape and their size was comparable to that measured with the dynamic light scattering (Supplementary Fig. S1). Dispersion was also sprayed onto adults and observed (Supplementary Fig. S2). No morphological modification of vesicles was observed after their application, confirming the maintenance of their structure after application (Supplementary Fig. S2). The microscopy observation indicated that geraniol-ethosomes filled the natural depressions of the eyes of the Mediterranean fruit fly (Supplementary Fig. S2). The abundant filling by nano-vesicles of the natural depressions presents in the eyes indicated a uniform cover over this biological surface^16,17^.

### In vitro evaluation of cytotoxic effects

Etofenprox is widely recognized as one of the safest pesticides for humans from a toxicological standpoint^51^. To conduct a preliminary safety assessment for agricultural operators and workers involved in field applications and re-entry activities (e.g., sorting, reaching, and picking), the acute toxicity of the formulations using human keratinocyte (HaCat) lines as representative skin surface cells, was assayed. The toxicity of each etofenprox formulation was evaluated at six different dilutions in the cell medium. Exposure to the commercial formulation caused significant cell toxicity, as the cell viability notably decreased in a concentration-dependent manner. The viability ranged from 49 ± 3% to 87 ± 3% at the highest (300 µg/mL) and the lowest (8.12 µg/mL) concentrations, respectively (Fig. 5). The high toxicity detected for the commercial formulation is not surprising, as numerous human health and environmental hazard pictograms are reported in the safety data sheet, due to the high amount (50–70%) of aromatic hydrocarbons, well known to induce oxidative stress and cell membrane damage^52^. The viability of cells treated with empty geraniol-ethosomes, diluted to match the concentrations used for the etofenprox-loaded phospholipid vesicles, ranged from 89 ± 5% at the lowest dilution to 107 ± 6% at the highest dilution (*p* > 0.05 compared to the viability of cells treated with the commercial formulation), demonstrating the high biocompatibility of the system (Fig. 5). Both ethosomes and geraniol-ethosomes showed a cell viability remarkably close to or above 100% at higher dilutions (32.5, 16.25, and 8.12 µg/mL) (*p* > 0.05 compared to control). At lower dilutions (300 and 150 µg/mL), cell viability was slightly lower but remained above 70%. Overall, the data confirmed the biocompatibility of ethosomes and geraniol-ethosomes, as they did not cause any cytotoxic effects. To our knowledge, no toxicity studies on keratinocytes involving etofenprox incorporated in nanocarriers are present in the literature. Similarly, Singh et al.^53^ found that chitosan alginate-cenosphere composite hydrogel beads loaded with imidacloprid were well tolerated by keratinocytes. Moreover, An et al.^54^ evaluated the impact of the ememectin benzoate loaded in a specifical tailored water-based nano delivery system on keratinocytes cells that did not cause a significant decrease in their viability compared to the emamectin benzoate granule, that in turn caused a reduction on cell viability at 250 mg/L.

## Conclusion

The aim of this work was to describe the preparation, physico-chemical characterization, and evaluation of the effectiveness of a new etofenprox formulation based on phospholipid vesicles (i.e., ethosome and geraniol-liposomes) against *C. capitata*. The one-step sonication method produced spherical and small phospholipid vesicles. The addition of geraniol significantly increased the stability of the formulation both at room temperature (over 90 days) and under thermal stress. The new formulations were compared with a commercial emulsifiable formulation. Regarding surface tension and foliar retention, ethosomes, geraniol-ethosomes, and the commercial formulation were comparable; however, after open-field application, geraniol-ethosomes left the highest etofenprox residues on lemon leaves (14.3 ± 1.0 mg/kg), whereas the commercial formulation on the flavedo (4.1 ± 0.5 mg/kg). This result highlighted the possibility of reducing the application dose of etofenprox loaded in geraniol-ethosomes especially during the BBCH (Biologische Bundesanstalt, Bundessortenamt and CHemical industry*)* stage before fruiting. Due to its slower release profile, geraniol-ethosomes were more effective against *C. capitata* than the commercial formulation in laboratory toxicity bioassays, achieving 100% mortality after 5 d. In contrast, about 80% of the active ingredient was released from the commercial formulation within one day, resulting in an overdosage in the first 24 h and an underdosage over the next 4 d (final mortality 55%). Due to its low surfactant composition, the geraniol-ethosomes formulation exhibited no toxic effects on human keratinocytes. Overall, the data obtained suggest that geraniol-ethosomes are a promising strategy for sustainable pest control in agriculture, according to recent policies and regulations on pesticide use.

## Supplementary Information

Supplementary Information.

## Data Availability

The datasets generated during and/or analysed during the current study are available from the corresponding author on reasonable request.
