# Supplementary material for: Design, characterization, and evaluation of eco-friendly etofenprox-loaded ethosomes to control Ceratitis capitata (Diptera: Tephritidae)
Source: Sci Rep. 2025 Jul 23;15:26699. doi: 10.1038/s41598-025-11832-y (PMC12284015; doi:10.1038/s41598-025-11832-y)
Supplement: Supplementary file 1 — Supplementary Information. [file 41598_2025_11832_MOESM1_ESM.docx]

**SUPPLEMENTARY MATERIALS**


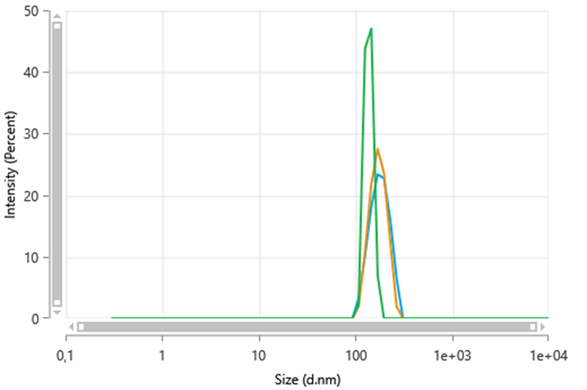


**Supplementary Figure S1**. Size distribution of the commercial formulation diluted following the label recommendation.

**
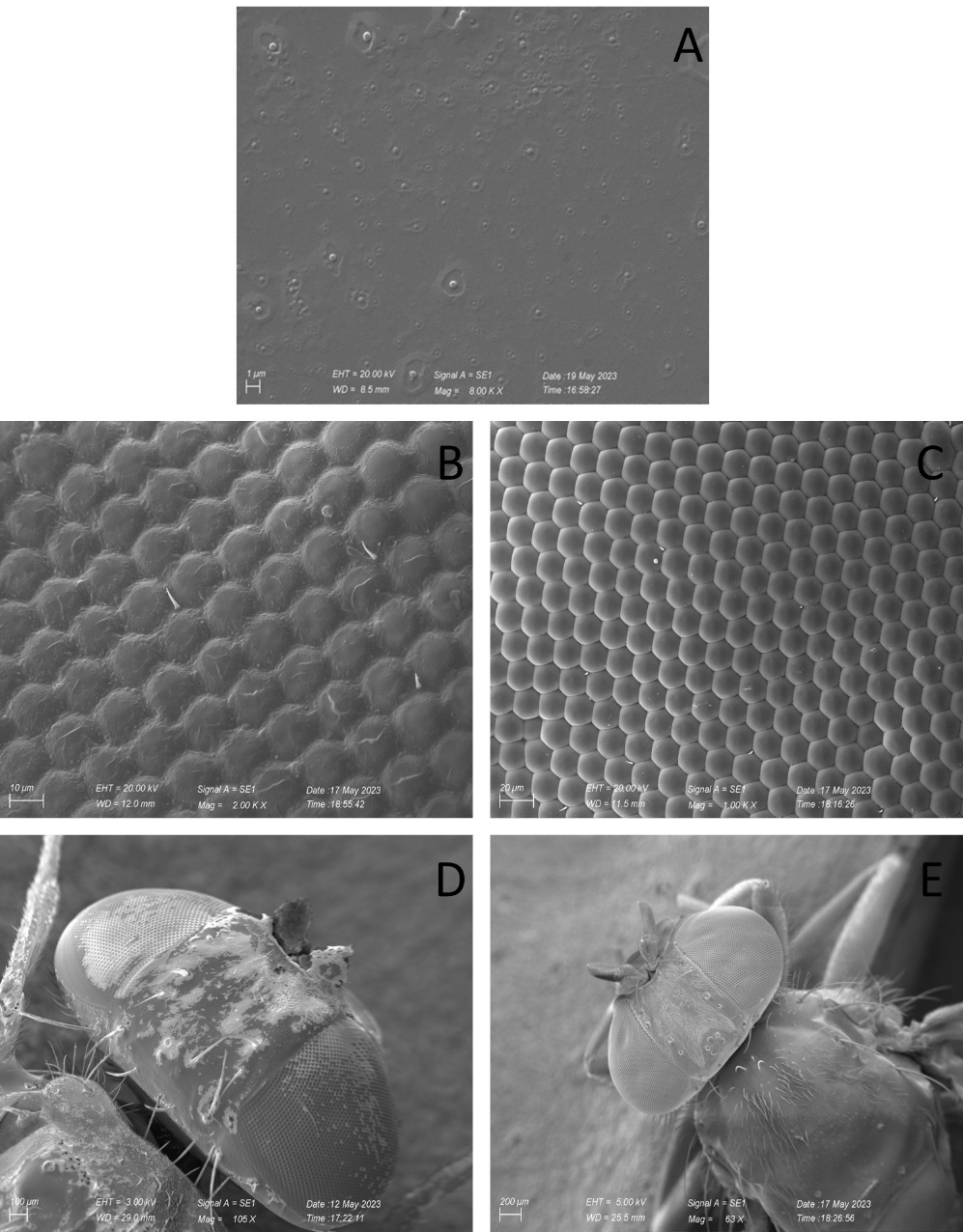
**

**Supplementary Figure S2.** Environmental scanning electron microscope images of diluted geraniol-ethosomes on the support surface (A); on the eyes of the Mediterranean fruit fly (B); on the head of the Mediterranean fruit fly (D); control image of Mediterranean fruit fly before application of geraniol-ethosomes (C and E).

**S1 Instrumental conditions**

**S1-1 UHPLC - QQQ**

A UHPLC Agilent 1290 Infinity II LC coupled with an Agilent 6470 Triple Quad LC-MS/MS mass detector with a MassHunter ChemStation was used. Briefly, a binary gradient composed of a 5 mM ammonium formate + 0.1% formic acid aqueous solution (A) and a 5 mM ammonium formate + 0.1% formic acid methanolic solution (B) was set as follows: t = 0 A 95%, t = 1 A 95%, t = 3.00 min A 55%, t = 16 min A 5%, t = 22.50 min A 5%, t = 22.60 min A 95%, with a post-run of 6 min (95% A), and with the total duration of the run being 28.60 min. One µL of sample volume was injected in positive mode. Mass detector gas and sheath-gas temperature were 120 ◦C and 325 ◦C, whereas sheath-gas flow, nebulizer, and positive capillary were set at 12 L min−1, 45 psi, and 3500 V, respectively. Data were acquired in Dynamic MRM. Mass transition 1: 394.2 – 107.1; mass transition 2: 394.2 – 177.2. Retention time: 17.2 minute.

**S1-2 HPLC – DAD**

Etofenprox quantification was carried out by using a HPLC Agilent 1100 series chromatograph equipped with a photodiode detector (DAD) and a computerized data integration system (ChemStation- Agilent). The column was a Phenomenex C18 (5 µm - 150 x 4.6 mm) working at room temperature. The analysis was carried out in isocratic condition with; the mobile phase consists of a binary solvent A (Acetonitrile) at 75% and B (MilliQ water) at 25% (total run time 12 minutes). The injection volume and the flow were set at 20 µL and 0.8 mL min -1, respectively. Etofenprox was evaluated at a fixed wavelength of 225 nm.
